# Association of single nucleotide polymorphisms in *IL8* and *IL13* with sunitinib-induced toxicity in patients with metastatic renal cell carcinoma

**DOI:** 10.1007/s00228-015-1935-7

**Published:** 2015-09-21

**Authors:** Meta H. M. Diekstra, Xiaoyan Liu, Jesse J. Swen, Epie Boven, Daniel Castellano, Hans Gelderblom, Ron H. J. Mathijssen, Cristina Rodríguez-Antona, Jesus García-Donas, Brian I. Rini, Henk-Jan Guchelaar

**Affiliations:** Department of Clinical Pharmacy and Toxicology, Leiden University Medical Center, Albinusdreef 2, Leiden, 2333ZA Netherlands; Dutch SUTOX consortium, Leiden, Netherlands; Institute of Clinical Pharmacology, Qilu Hospital of Shandong University, Jinan, China; Department of Medical Oncology, VU University Medical Center, Amsterdam, Netherlands; Oncology Department, Hospital Universitario 12 de Octubre, Madrid, Spain; Spanish Oncology Genitourinary Group (SOGUG), Madrid, Spain; Department of Medical Oncology, Leiden University Medical Center, Leiden, Netherlands; Department of Medical Oncology, Erasmus MC Cancer Institute, Rotterdam, Netherlands; Hereditary Endocrine Cancer Group, Spanish National Cancer Research Centre (CNIO), Madrid, Spain; ISCIII Center for Biomedical Research on Rare Diseases (CIBERER), Madrid, Spain; Oncology Unit, Clara Campal Comprehensive Cancer Center, Madrid, Spain; Department of Solid Tumor Oncology, Cleveland Clinic Taussig Cancer Institute (CCF), Cleveland, OH USA

**Keywords:** Tyrosine kinase inhibitor, Sunitinib, Metastatic renal cell carcinoma, Single nucleotide polymorphism, Toxicity, Progression-free survival

## Abstract

**Purpose:**

Earlier, the association of single nucleotide polymorphisms (SNPs) with toxicity and efficacy of sunitinib has been explored in patients with metastatic renal cell carcinoma (mRCC). Recently, additional SNPs have been suggested as potential biomarkers. We investigated these novel SNPs for association with sunitinib treatment outcome in mRCC patients.

**Methods:**

In this exploratory study, we selected SNPs in genes *CYP3A4*, *NR1I2*, *POR*, *IL8*, *IL13*, *IL4-R*, *HIF1A* and *MET* that might possibly be associated with sunitinib treatment outcome. Each SNP was tested for association with progression-free survival (PFS) and overall survival (OS) by Cox-regression analysis and for clinical response and toxicity using logistic regression.

**Results:**

We included 374 patients for toxicity analyses, of which 38 patients with non-clear cell renal cell cancer were excluded from efficacy analyses. The risk for hypertension was increased in the presence of the T allele in *IL8* rs1126647 (OR = 1.69, 95 % CI = 1.07–2.67, *P* = 0.024). The T allele in *IL13* rs1800925 was associated with an increase in the risk of leukopenia (OR = 6.76, 95 % CI = 1.35–33.9, *P* = 0.020) and increased prevalence of any toxicity > grade 2 (OR = 1.75, 95 % CI = 1.06–2.88, *P* = 0.028). No significant associations were found with PFS, OS or clinical response.

**Conclusions:**

We show that polymorphisms in *IL8* rs1126647 and *IL13* rs1800925 are associated with sunitinib-induced toxicities. Validation in an independent cohort is required.

**Electronic supplementary material:**

The online version of this article (doi:10.1007/s00228-015-1935-7) contains supplementary material, which is available to authorized users.

## Introduction

Several targeted therapies have been approved for the treatment of metastatic renal cell carcinoma (mRCC). Of these, the tyrosine kinase inhibitor (TKI) sunitinib is widely used as first-line treatment option [1–4]. The response to sunitinib varies largely among patients. Only 35 % of mRCC patients benefit from sunitinib, and about 30 % of patients need dose reductions due to adverse events of which grades vary among patients [1, 5, 6]. To optimize treatment efficacy and to minimize the risk of adverse events of higher grades, it would be helpful to predict the individual treatment outcome at the initiation of therapy. Unfortunately, no biomarkers are yet available to fulfil this need.

Several studies used a candidate gene approach to select single nucleotide polymorphisms (SNPs) in genes involved in the pharmacokinetics and pharmacodynamics of sunitinib. SNPs and haplotypes in *CYP1A1*, *CYP3A5*, *ABCB1*, *ABCG2*, *NR1I3*, *VEGFA*, *NOS3* (=*eNOS*), *FLT1* (*=VEGFR1*), *KDR* (=*VEGFR2*), *FLT-4* (=*VEGFR3*) and *FLT3* have been described to have an association with either toxicity or efficacy of sunitinib (*P* < 0.05) in mRCC patients [5–11]. The associations of *CYP3A5* and *ABCB1* with dose reductions and efficacy, respectively, have been confirmed recently [12]. Not all clinical outcomes can be explained by these potential biomarkers, which make identification of other markers possibly associated with clinical outcome an attractive prospect.

Novel SNPs in *CYP3A4*, *NR1I2*, *POR*, *IL8*, *IL4-R*, *IL13*, *HIF1A* and *MET* have been reported in patients with RCC, either prognostic or associated with treatment outcome, and might play a role in sunitinib treatment outcome (Table 1) [13–20]. In some studies on these novel SNPs, the TKI pazopanib was given to patients with mRCC. Sunitinib and pazopanib have similar efficacy and both are used as first-line treatment options [2]. Further, both drugs have similarities in their metabolic pathways and affected targets, because of which SNPs associated with pazopanib outcome might also be meaningful for the sunitinib treatment outcome, i.e. toxicity or efficacy [21]. The T allele of SNP rs35599367 in *CYP3A4* (CYP3A4*22) was associated with decreased clearance of sunitinib [13–15]. The T allele of SNP rs3814055 in *NR1I2* was associated with a reduction in response to pazopanib and inferior progression-free survival (PFS) from sunitinib and pazopanib in univariate analysis [6, 16]. The TT genotype of SNP rs1057858 in the P450 oxidoreductase gene (POR*28) was associated with higher CYP3A activity [17, 18]. Variant alleles of rs4073 and rs1126647 in *IL8* were associated with an inferior PFS on pazopanib treatment [16]. SNPs rs1800925 and rs20541 in *IL13* and rs180510 in *IL4-R* are likely to influence tumour immune response and carcinogenesis [19]. The AG genotype of rs11549467 in *HIF1A* compared to wild-type GG was associated with a decreased PFS and a reduced response rate on pazopanib treatment [16]. The A-allele of SNP rs11762213 in *MET* was associated with an increased risk of recurrence or death in RCC patients [20].

**Table 1:** Polymorphisms in candidate genes in the current study. Genetic polymorphisms were included if in previous exploratory studies associations were reported with a *P* value <0.05

Gene	SNP	Reported results	References
*CYP3A4*	rs35599367C>T	CYP3A4*22 is associated with decreased CYP3A4 expression and decreased clearance of sunitinib.	Elens et al. [13, 14] Diekstra et al. [15]
*NR1I2*	rs3814055C>T	T allele is associated with reduction of response to pazopanib and inferior PFS on sunitinib and pazopanib.	van der Veldt et al. [6] Xu et al. [16]
POR*28	rs1057868C>T	POR*28 is associated with higher in vivo CYP3A activity.	de Jonge et al. [17] Oneda et al. [18]
*IL8*	rs4073T>A	AA shows inferior PFS of pazopanib compared to wild-type TT.	Xu et al. [16]
	rs1126647A>T	TT shows inferior PFS of pazopanib compared to wild-type AA.	Xu et al. [16]
	Correlated SNPs rs4073T>A and rs1126647A>T	Inferior PFS for variant genotypes (r4073 AA + rs1126647 TT) compared to wild types (rs4073 TT + rs1126647 AA)	Xu et al. [16]
*HIF1A*	rs11549467G>A	AG genotype is associated with inferior PFS and reduced response rate of pazopanib compared to wild-type GG.	Xu et al. [16]
*IL4-R*	rs1805010T>C	CT/TT genotype is associated with decreased risk of RCC compared to CC genotype.	Chu et al. [19]
*IL13*	rs1800925C>T	TT genotype is associated with decreased risk of RCC.	Chu et al. [19]
	rs20541C>T		
*MET*	rs11762213G>A	A-allele is associated with an increased risk of recurrence or death.	Schutz et al. [20]

*SNP* single nucleotide polymorphism, *PFS* progression-free survival, *OS* overall survival

In this exploratory study, we evaluated the polymorphisms in the above-mentioned genes for possible associations with toxicity or efficacy of sunitinib in a large cohort of mRCC patients.

## Methods

### Study population

Patient data were collected from three exploratory studies (SUTOX, SOGUG and CCF) between the years 2004 and 2010 (Supplementary document 1) [12]. SUTOX samples were anonymized by a third party, according to the instructions stated in the Codes for Proper Use and Proper Conduct in the Self-Regulatory Codes of Conduct (www.federa.org). The study was conducted in accordance with the Declaration of Helsinki and approved by the medical ethics review board of all participating groups. Patients provided their written informed consent for participation [12].

### Study endpoints

PFS, defined as the time in months between the first day of sunitinib treatment and the date of progressive disease (PD) according to Response Evaluation Criteria in Solid Tumours (RECIST) v.1.0 or v1.1, was used as the primary endpoint to assess efficacy. Another endpoint was overall survival (OS), which was measured from the first day of sunitinib treatment until death or time of last follow-up.

We classified objective clinical response into three categories: (i) partial and complete response, (ii) stable disease and (iii) progressive disease (according to RECIST).

Specific sunitinib-related adverse events, i.e. thrombocytopenia, leukopenia, mucosal inflammation, hand-foot syndrome, hypertension and any toxicity > grade 2, were collected for this study (Supplementary document 1) [12].

### Statistical analysis

For univariate analysis, a log-rank test was used for the association of each SNP with PFS and OS, and a chi-square test for clinical response and toxicity. SNPs with a *P* value <0.1 were included in the multivariate model. Based on previous results, we included well-established covariates age, gender and Heng prognostic risk group in the multivariate model for correction of PFS and OS [22]. In addition, the CGT haplotype of *ABCB1* was also used as covariate in multivariate Cox model because of the previously confirmed significant association with PFS [6, 12]. For multivariate analysis of clinical response, we also included age, gender and Heng prognostic risk group as covariates. For multivariate analysis on toxicity endpoints, no biomarker was widely validated, so we corrected for age and gender. Because this association study used data from three study groups (SUTOX, SOGUG and CCF), study group was also tested as a covariate for all endpoints. It was not needed to correct for previous treatment in efficacy analyses, because this was already justified by using study group as a covariate.

Reported results from the multivariate analysis with a *P* value <0.05 were considered clinically significant. All tests were two-sided and carried out by SPSS Statistical Package for Windows (version 20.0 Armonk, NY: IBM Corp).

## Results

### Patient characteristics

**Fig. 1:**
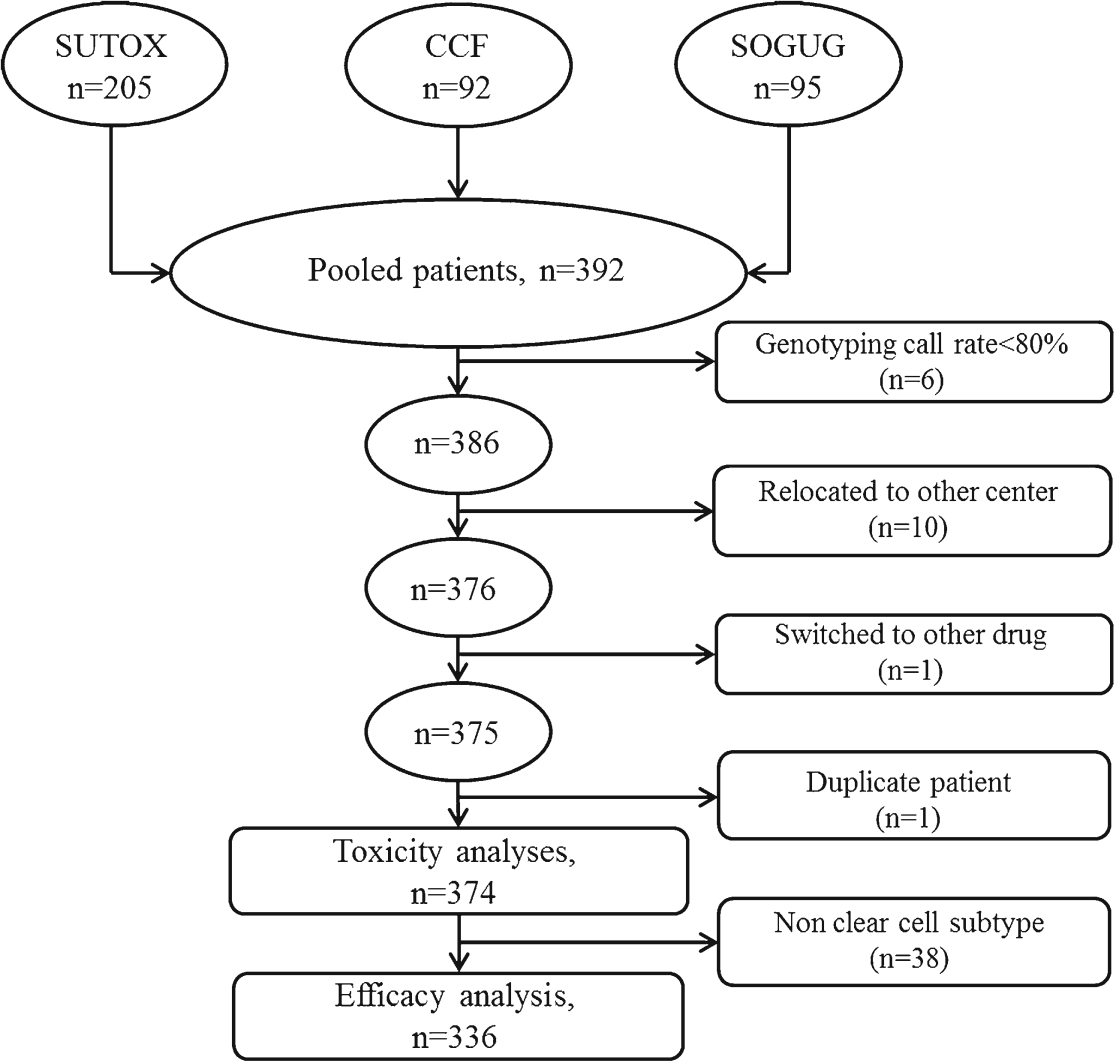
Patient flowchart on included patients. Fifty-six patients had to be excluded from association analyses because of individual genotyping call rates <80 % (*N* = 6), relocation to another medical center during follow-up (*N* = 10), double patient (*N* = 1), a change to another treatment than sunitinib directly after enrolment (*N* = 1) or non-clear cell subtypes (*N* = 38). A total of 374 sunitinib-treated clear cell mRCC patients were available for analysis of toxicity in the present study. For efficacy analysis, 336 sunitinib-treated clear cell mRCC patients were available [12]

A total of 374 patients with mRCC treated with sunitinib were included for association analyses on toxicity endpoints. For efficacy analyses, only clear cell subtypes (*N* = 336) were included (Fig. 1). Patient characteristics are presented in Table 2. Median age of patients was 61 years, and most were men of Caucasian ethnicity. The majority of patients had undergone nephrectomy. For the clear cell subjects, the good or intermediate Heng prognostic risk group consisted of 73 % of the patients. Forty-five percent (*N* = 143) of patients showed a partial response (PR) or complete response (CR) to sunitinib.

**Table 2:** Patient characteristics

Characteristic	Efficacy cohort (*N* = 336)	Toxicity cohort (*N* = 374)
Age		
Median	61	61
Percentiles (25th, 75th)	55, 69	54, 68
BSA		
Median	1.98	1.96
Percentiles (25th, 75th)	1.82, 2.13	1.82, 2.12
Race		
White	324 (96 %)	358 (96 %)
Asian	3 (0.89 %)	3 (0.80 %)
Black	5 (1.5 %)	8 (2.1 %)
Arabian	3 (0.89 %)	4 (1.0 %)
Latin American	1 (0.30 %)	1 (0.27 %)
Gender		
Male	229 (68 %)	258 (69 %)
Female	107 (32 %)	116 (31 %)
Clinical response		
PR + CR	143 (43 %)	149 (40 %)
SD	130 (39 %)	149 (40 %)
PD	43 (13 %)	55 (15 %)
Unknown	20 (6.0 %)	21 (5.6 %)
Heng prognostic risk group^a^		
Good (0 risk factor)	64 (19 %)	68 (18 %)
Intermediate (1–2 risk factors)	182 (54 %)	197 (53 %)
Poor (3–6 risk factors)	90 (27 %)	108 (29 %)
Metastatic sites		
1	91 (27 %)	105 (28 %)
2	137 (41 %)	149 (40 %)
≥3	104 (31 %)	116 (31 %)
Prior nephrectomy		
No	49 (15 %)	63 (17 %)
Yes	283 (85 %)	307 (82 %)
Prior treatment		
No	254 (76 %)	276 (74 %)
Yes	82 (24 %)	98 (26 %)

*BSA* body surface area, *CR* complete response, *PR* partial response, *SD* stable disease, *PD* progressive disease

^a^Patients are classified according to the six Heng risk criteria: poor WHO performance status (≥2), low haemoglobin (<lower limit of normal), high calcium (>upper limit of normal) and time from initial diagnosis to treatment with sunitinib (<1 year), neutrophil count (>upper limit of normal) and thrombocytes (>upper limit of normal)

95.7 to 99.5 % of patients had no toxicities at baseline. Within four cycles of sunitinib treatment, any grade of thrombocytopenia was observed in 61 % of patients, mucosal inflammation in 59 %, leukopenia in 49 %, hand-foot syndrome in 41 % and hypertension in 38 %. Twenty-six percent of patients developed any toxicity > grade 2 (Supplementary Table S1).

For clear cell subtypes, median follow-up times for PFS and OS analysis were 43 and 49 months, respectively. Median PFS and OS of patients were 16 and 26 months, respectively.

### Genotyping

*IL*8 rs4073 was excluded from statistical analysis because the call rate was less than 95 %. The SNP genotype call rate for the remaining SNPs ranged from 97.6 to 99.7 %. All SNPs were in Hardy-Weinberg equilibrium (HWE) (*P* > 0.05 and *χ*^2^ < 3.84). The allele frequencies of genotyped polymorphisms were similar as reported in the National Center for Biotechnology Information (NCBI) SNP database [23]. Only three patients with the *HIF1A* rs11549467 heterozygous genotype were detected. As a consequence, the effects of this SNP could not be analysed.

### Genetic association analysis

No significant associations between SNPs and PFS, OS or objective response to sunitinib were observed in this study. Although not significant, A-allele carriers of *MET* rs11762213 had a better PFS compared to wild-type GG (HR = 0.63, 95 % CI = 0.38–1.05, *P* = 0.076), adjusted for age, gender, Heng prognostic risk group, study group and CGT haplotype in *ABCB1* (Table 3).

**Table 3:** Univariate and multivariate analyses for SNPs associated with sunitinib treatment outcome

Treatment outcome	Number of patients	Univariate analyses			Multivariate analyses		
		HR or OR	95 % CI	*P* value	HR or OR	95 % CI	*P* value
PFS							
*MET* rs11762213 G>A		0.58	0.36–0.96	0.032	0.63	0.38–1.05	0.076^a^
GG versus	302						
AG+AA	33						
Hypertension							
*IL8* rs1126647 A>T		1.70	1.08–2.68	0.022	1.69	1.07–2.67	0.024^b^
AA versus	128						
AT versus	174						
TT	61						
Thrombocytopenia							
*IL13* rs20541 C>T		1.97	0.90–4.35	0.092	1.87	0.82–4.23	0.136^b^
CC versus	235						
CT+TT	117						
Leukopenia							
*IL13* rs1800925 C>T		6.13	1.25–30.0	0.025	6.76	1.35–33.9	0.020^b^
CC versus	232						
CT+TT	137						
Any toxicity > grade 2							
*IL13* rs1800925 C>T		1.91	1.15–3.15	0.012	1.75	1.06–2.88	0.028^b^
CC versus	234						
CT+TT	137						

*HR* hazard ratio, *OR* odds ratio, *CI* confidence interval, *PFS* progression-free survival

^a^Adjusted by age, gender, Heng prognostic risk group, study center and *ABCB1* haplotype

^b^Adjusted by age, gender and study center

In multivariate toxicity analysis, the T allele of *IL8* rs1126647 was associated with an increased risk of hypertension compared to wild-type AA (OR = 1.69, 95 % CI = 1.07–2.67, *P* = 0.024) after adjustment by age, gender and study group. Presence of the *IL13* rs1800925 T allele was associated with an increased risk of leukopenia (OR = 6.76, 95 % CI = 1.35–33.9, *P* = 0.020) and also with development of any toxicity > grade 2 (OR = 1.75, 95 % CI = 1.06–2.88, *P* = 0.028) (Table 3).

## Discussion

In the present study, we analysed eight candidate SNPs in seven genes for potential association with response and/or toxicity of sunitinib in a large cohort of mRCC patients. To our knowledge, we report for the first time that genetic polymorphisms in interleukin genes *IL8* and *IL13* are associated with the occurrence of clinically relevant adverse events from sunitinib. Patients carrying a T allele of *IL8* rs1126647 had an increased risk of hypertension compared to wild-type AA (OR = 1.69). T carriers of *IL13* rs1800925 had an increased risk of leukopenia (OR = 6.76) and an increased risk of any toxicity > grade 2 (OR = 1.75).

Hypertension is a frequent toxicity caused by TKIs. The potential mechanism is suggested to be related to inhibition of VEGFR-2 and decrease of nitric oxide, resulting in vasoconstriction and elevated blood pressure [8, 24]. It has been recognized that IL8, possibly by upregulating VEGF levels through NFkappaB [25, 26], can play a role in stimulating VEGFR-2 transactivation. In our data, presence of the variant allele in *IL8* rs1126647A>T is significantly associated with hypertension. *IL8* rs1126647A>T is in linkage with *IL8* rs4073T>A (*r*^2^ = 0.78) [16]. It was shown that a haplotype of *IL8*, including the variant allele of rs4073T>A and rs1126647A>T, was associated with increased IL8 expression [27]. In contrast, Amaya et al. [28] have reported that the variant allele of rs4073 was associated with a lower production of IL8. It remains to be elucidated whether particular SNPs in *IL8* have prognostic or even predictive significance in metastatic renal cell carcinoma.

The occurrence of hypertension is a well-known predictor for increased survival in metastatic renal cell carcinoma treated with a TKI [29]. In addition to an increased occurrence of hypertension, patients with the variant allele in *IL8* rs1126647A>T showed a trend towards improved PFS/OS (22 vs 14 months for median PFS and 40 vs 25 months for median OS), although the differences between groups did not reach statistical significance. One possible reason for the lack of statistical significance in the above association may be the ∼2 fold larger (*n* = 544) sample size of the study from Rini et al. [29]. Xu et al. [16] described an inferior PFS of pazopanib for carriers of the variant allele of *IL8* rs1126647A>T. In a follow-up study, Xu et al. [30] reported that the variant allele of *IL8* rs1126647A>T was significantly associated with worse OS in mRCC patients treated with pazopanib or sunitinib, but could not confirm the initial association with PFS. Unfortunately, Xu et al. do not report data on associations between *IL8* rs1126647 and hypertension. In addition, many factors can have an impact on OS, including baseline conditions and previous and posterior treatments. Therefore, it is not possible to directly compare the association of *IL8* rs1126647 and hypertension/survival between the two datasets and further study in an independent cohort is required.

We observed an increased risk of leukopenia in carriers of the variant allele of *IL13* rs1800925C**>**T. Leukopenia is a common haematologic adverse event in sunitinib treatment of which the mechanism remains uncertain. It was reported that VEGF and its receptors (VEGFR) are essential for development of aberrant haematopoiesis, including leukopenia [31]. Shen et al. [32] have reported that VEGFR-2 can be upregulated by IL13. The T allele of *IL13* rs1800925 has been found to be associated with increased IL13 protein function [33].

Little is known about the effects of SNPs in *IL8* and *IL13*, but it is likely that these SNPs will either increase or decrease the protein expression of IL8 and IL13 proteins [24–26, 29, 31–34]. It also remains unclear whether the function of these interleukins will affect TKI treatment outcome and if and how these interleukin proteins would influence the VEGF(R) pathway directly or indirectly [25–28, 30–36]. Xu et al. [30] speculate that patients with the *IL8* variants having a high IL8 expression may have more aggressive tumours, and therefore a reduced survival. However, it is difficult to hypothesize because we noticed conflicting results: Hacking et al. [27] reported increased expression for *IL8* variants, while a more recent paper of Amaya et al. [28] reported the opposite effect on IL8 expression. If variant alleles in *IL8* and *IL13* SNPs alter protein expression and have an effect on VEGF-R2, a stronger inhibition of VEGF-R2 in the concomitant presence of sunitinib would explain our results on a higher risk for any toxicity, including hypertension and leukopenia.

HIF1A protein regulates the transcription of a large number of genes that respond to hypoxia, among which is angiogenesis. The A-allele frequency of *HIF1A* rs11549467 in our cohort was 0.45 %, which is lower than that (*A* = 3.0 %) reported by Xu et al. [16], but corresponds with the result (*A* = 0.6 %) from Beuselinck et al. [10]. The difference in A-allele frequency is potentially caused by the subject selection. Of note, the subjects recruited here and in the study of Beuselinck typically have Western European ancestry, whereas the patients in Xu’s study are mostly from Eastern Europe [10, 16].

This study was conducted in a relatively large cohort, which decreases the chance for false-positive findings. In addition to Xu et al. [30], we have tested on toxicity as well as efficacy endpoints, which is essential for clinical interpretation. SNPs could influence the drug exposure to the TKI and consequently have an effect on both adverse events, PFS and OS. Because this study only presents explorative findings without external validation, and our results are based on mainly Caucasian subjects, extrapolation to the entire mRCC population is difficult.

To capture adverse events that occur later on in treatment, all toxicity outcomes were recorded and evaluated up to four cycles of sunitinib treatment. In our cohort, hypertension and fatigue have a large contribution in the endpoint any toxicity > grade 2. Because it is difficult to evaluate fatigue objectively, we did not test fatigue as separate toxicity endpoint. Since our study represents data collected from 2004 to 2010, it is likely that clinical practice (i.e. toxicity management) has evolved in the course of time. In earlier years with limited experience on sunitinib treatment, dose reductions were applied in the case of grade 3 or 4 toxicities. Nowadays, physicians anticipate on the development of severe adverse events by already reducing the dose on the occurrence of grade 2 toxicity. However, we did not observe a difference in dose reductions between earlier years and later years (data not shown). Further, our dataset lacks sufficiently detailed information on the reasons and time points of dose reductions.

Our SNP selection was based on reported associations with toxicity and efficacy outcomes on treatment and the risk of developing RCC. SNPs are often categorized as being either predictive or prognostic biomarkers. However, this distinction may not have to be as rigorous as we currently assume. In fact, prediction and prognosis may be strongly correlated. Antitumour treatment can have a different effect in patients with a more aggressive tumour type that is possibly caused by an underlying SNP. For example, high IL8 protein levels in RCC are considered to have a poor prognosis [35]. However, elevated IL8 levels investigated in preclinical models resulted in resistance to sunitinib, which would be considered predictive [36]. Functional studies on immunological and angiogenic factors and a genome-wide approach can help us in understanding the mechanisms on the predictive or prognostic character of the SNPs.

In conclusion, this study suggests a relationship between interleukin genes *IL8* and *IL13* and the development of sunitinib-induced adverse events. Further validation in an independent cohort is warranted to confirm our findings. In addition, we consider functional studies on IL8 and IL13, with respect to regulation of VEGF(R) genes and sVEGF(R) plasma levels, to be crucial for our understanding of the mechanisms involved in sunitinib exposure and occurrence of adverse events.

## Electronic supplementary material

Supplementary Table S1 (DOC 36 kb)

Supplementary document 1 (DOCX 15 kb)
