# Supplementary material for: Association of single nucleotide polymorphisms in IL8 and IL13 with sunitinib-induced toxicity in patients with metastatic renal cell carcinoma
Source: Eur J Clin Pharmacol. 2015 Sep 21;71(12):1477–84. doi: 10.1007/s00228-015-1935-7 (PMC4643117; doi:10.1007/s00228-015-1935-7)
Supplement: Supplementary file 1 — (DOC 36 kb) [file 228_2015_1935_MOESM1_ESM.doc]

**Supplementary Table S1**. Distribution of most common adverse events within four cycles of sunitinib treatment

| **Toxicity by grade** | **No. of patients (*N*)** | **Percentage of patients (%)** |
| --- | --- | --- |
| **Fatigue**  0  1  2  3  4 | 118  117  100  37  2 | 32  31  27  9.9  0.50 |
| **Thrombocytopenia**  0  1  2  3  4 | 145  148  49  28  4 | 39  40  13  7.5  1.1 |
| **Mucosal inflammation**  0  1  2  3 | 154  123  75  22 | 41  33  20  5.9 |
| **Leukopenia**  0  1  2  3 | 189  106  67  12 | 51  28  18  3.2 |
| **Hand-foot-syndrome**  0  1  2  3 | 222  69  57  26 | 59  18  15  7.0 |
| **Hypertension**  0  1  2  3 | 231  37  55  51 | 62  10  15  14 |
| **Diarrhoea**  0  1  2  3 | 227  90  44  13 | 61  24  12  3.5 |
| **Any toxicity > grade 2**  0, 1, 2  3, 4 | 227  97 | 74  26 |
