# Supplementary material for: Association of single nucleotide polymorphisms in IL8 and IL13 with sunitinib-induced toxicity in patients with metastatic renal cell carcinoma
Source: Eur J Clin Pharmacol. 2015 Sep 21;71(12):1477–84. doi: 10.1007/s00228-015-1935-7 (PMC4643117; doi:10.1007/s00228-015-1935-7)
Supplement: Supplementary file 2 — (DOCX 15 kb) [file 228_2015_1935_MOESM2_ESM.docx]

**Appendix A – Additional methods**

**Study Population**

The SUTOX consortium includes five medical centers in the Netherlands, the Spanish Oncology Genitourinary Group (SOGUG) consists of fifteen Spanish medical centers, and CCF represents the Taussig Cancer Institute of Cleveland Clinic Foundation in the United States. Patient characteristics with details on treatment, efficacy and toxicity were retrieved from the individual records. Sunitinib was administered orally either as first or second line treatment at a starting dose of 50mg, 37.5mg or 25mg daily, generally given as a 4-weeks-on and 2-weeks-off treatment cycle or as a continuous dosing regimen. Dose modification or dose delay was done for higher grades of adverse events. Eligible patients for the current study received sunitinib for at least one cycle of 6 weeks, and had a blood sample available for DNA extraction.

Genotyping

Germline DNA was extracted from whole blood, serum, plasma or peripheral blood mononuclear cell samples. Based on previous studies, we selected ten polymorphisms in genes *CYP3A4*, *NR1I2*, *POR*, *IL8*, *HIF1A*, *IL13*, *IL4-R* and *MET* (**Table 1**), which might be associated with sunitinib treatment [6,13-20]. Full genotyping methods are described in our previous study [12]. SUTOX and CCF samples were genotyped at Leiden University Medical Center (LUMC) using Taqman probes (Applied Biosystems, Nieuwerkerk aan den IJssel, the Netherlands) on the LightCycler480 (LC480) Real-Time PCR Instrument (Roche Applied Science, Almere, The Netherlands). SOGUG samples were genotyped by the Spanish National Cancer Research Centre (CNIO) using a KASPar SNP genotyping system (Kbiosciences, Hoddesdon, UK) and the sequence Detection System 7900HT (Applied Biosystems, Foster City, CA, USA) for fluorescence detection and allele assignment.

To exclude inter assay variation between Leiden University Medical Center (LUMC) and Spanish National Cancer Research Centre (CNIO), a selection of 132 anonymized SOGUG DNA samples were re-genotyped in Leiden. A 98% concordance in SNP genotypes was observed between the LUMC and CNIO samples [12].

**Study Endpoints**

Patients with unknown response status were excluded from response analysis. Cumulative toxicity for the first four treatment cycles was corrected by baseline conditions, and was used as dichotomous groups, grade 0-2 and grade 3-4 [12].

Adverse events were evaluated at baseline, after 4 weeks and 6 weeks in each cycle using the National Cancer Institute Common Terminology Criteria for Adverse Events v.3.0 or v.4.0 [12].

**Statistical analysis**

We fitted dominant and additive genetic models in this study, depending on the distribution of genotypes of each SNP (common homozygous, heterozygous and variant homozygous).
